# Modulation of Cell Signaling Networks after CTLA4 Blockade in Patients with Metastatic Melanoma

**DOI:** 10.1371/journal.pone.0012711

**Published:** 2010-09-15

**Authors:** Begoña Comin-Anduix, Hooman Sazegar, Thinle Chodon, Douglas Matsunaga, Jason Jalil, Erika von Euw, Helena Escuin-Ordinas, Robert Balderas, Bartosz Chmielowski, Jesus Gomez-Navarro, Richard C. Koya, Antoni Ribas

**Affiliations:** 1 Division of Surgical Oncology, Department of Surgery, University of California Los Angeles, Los Angeles, California, United States of America; 2 Jonsson Comprehensive Cancer Center, University of California Los Angeles, Los Angeles, California, United States of America; 3 Division of Hematology/Oncology, Department of Medicine, University of California Los Angeles, Los Angeles, California, United States of America; 4 BD Biosciences, San Jose, California, United States of America; 5 Pfizer Global Research and Development, New London, Connecticut, United States of America; Universität Würzburg, Germany

## Abstract

**Background:**

The effects on cell signalling networks upon blockade of cytotoxic T lymphocyte-associated antigen-4 (CTLA4) using the monoclonal antibody tremelimumab were studied in peripheral blood mononuclear cell (PBMC) samples from patients with metastatic melanoma.

**Methodology/Principal:**

Findings Intracellular flow cytometry was used to detect phosphorylated (p) signaling molecules downstream of the T cell receptor (TCR) and cytokine receptors. PBMC from tremelimumab-treated patients were characterized by increase in pp38, pSTAT1 and pSTAT3, and decrease in pLck, pERK1/2 and pSTAT5 levels. These changes were noted in CD4 and CD8 T lymphocytes but also in CD14 monocytes. A divergent pattern of phosphorylation of Zap70, LAT, Akt and STAT6 was noted in patients with or without an objective tumor response.

**Conclusions/Significance:**

The administration of the CTLA4-blocking antibody tremelimumab to patients with metastatic melanoma influences signaling networks downstream of the TCR and cytokine receptors both in T cells and monocytes. The strong modulation of signaling networks in monocytes suggests that this cell subset may be involved in clinical responses to CTLA4 blockade.

**Clinical Trial Registration:**

clinicaltrials.gov; Registration numbers NCT00090896 and NCT00471887

## Introduction

The cytotoxic T lymphocyte associated antigen 4 (CTLA4, CD152) is an activation-induced immunoglobulin family receptor expressed by T lymphocytes that provides a dominant negative signaling upon binding to the costimulatory molecules CD80 (B7.1) and CD86 (B7.2), leading to T cell tolerance and anergy [1]. Expression of CTLA4 on T cells is tightly regulated. In naïve T cells, surface CTLA4 expression is inhibited by rapid endocytosis resulting from CTLA4 binding to AP50, a subunit of the clathrin adaptor AP-2 protein [2]. Once a T cell is activated through the T cell receptor (TCR), downstream TCR signaling through src kinases results in tyrosine phosphorylation of CTLA4 and uncoupling it from AP50, resulting in its surface expression with a peak of 48 hours after activation. Because of its much higher affinity for B7 costimulatory molecules, cell surface CTLA4 efficiently competes with the positive costimulatory receptor [1]. The engagement of CTLA4 results in decreased TCR signaling, decreased interleukin 2 (IL-2) transcription [3], and cell cycle arrest at the G1 to S transition [4], [5]. The phenotype of CTLA4 knock out mice, which develop massive T cell proliferation and autoimmune infiltration of multiple organs within weeks after birth, provides evidence of the critical role of CTLA4 in regulation of immune responses [6], [7]. In addition, CTLA4 has been detected on the surface of monocytes, but its role is currently not fully understood [8].

Tremelimumab (formerly CP-675,206) is a fully human IgG2 monoclonal antibody in clinical development for patients with cancer [9]. Clinical trials using tremelimumab demonstrate that this antibody can induce durable tumor regressions (up to 8 years at this time) in 7 to 10% of patients with metastatic melanoma [10], [11]. The presence of activated cytotoxic T lymphocytes (CTLs) against melanoma has been confirmed by serial analysis of patient-derived tumor biopsies [12]. Grade 3 or 4 toxicities in the range of 20–30% are mainly inflammatory or autoimmune in nature, which are on-target effects after inhibiting CTLA4-mediated self-tolerance [10], [11]. The lack of survival advantage in the early analysis of a phase III clinical trial comparing tremelimumab with standard chemotherapy for metastatic melanoma highlights the importance of a better understanding of how this antibody modulates the human immune system [13]. Therefore, we studied changes in signaling pathways downstream of the TCR and cytokine receptors, two major signaling pathways modulated by CTLA4 blockade [3], [14], [15], in PBMC from patients treated with tremelimumab.

The inhibitory effect of triggering CTLA4 by costimulatory molecules results in direct inhibition of signaling downstream of the TCR/CD3 complex [3]. A direct association between CTLA4 with CD3ζ leading to its dephosphorylation has been reported, resulting in the inhibition of of the leukocyte-specific protein tyrosine kinase (Lck), a member of the src family of tyrosine kinases responsible for early downstream signaling from the TCR [3]. The inhibitory role of CTLA4 on TCR signaling is evident when analyzing lymphocytes from mice genetically deficient in CTLA4, which demonstrate markedly increased phosphorylation of CD3ζ-chain-associated protein kinase 70 (ZAP70), SHC, Fyn and Lck [14]. Further confirmatory evidence for the critical role of CTLA4 in inhibiting downstream signaling from the TCR comes from studies in humans with a polymorphism in the CTLA4 gene region known as CT60 (A/G), which results in increased incidence of autoimmune diseases like type 1 diabetes, Graves disease and Addison’s disease [16]. CD4^+^ T cells from subjects with this polymorphism have increased ability to phosphorylate downstream molecules upon CD3 stimulation [17]. TCR/CD3 signaling and src kinase family phosphorylation leads to the activation of Ras and further downstream activation of the mitogen-activated protein kinase family (MAPK), including the extracellular signal-regulated kinase (ERK), the Jun N-terminal kinase (JNK) and the p38 MAPK, a pathway that leads to T cell proliferation [18]. Its inhibition by CTLA4 engagement on T cells results in a cell cycle arrest, with inhibition at the G1 stage [4], [5]. The cell cycle arrest induced by CTLA4 is not universal, since this effect varies in different lymphocyte subsets and is dependent on prior cognate antigen exposure. The CTLA4-induced cell cycle arrest is particularly evident in CD4^+^ T cells upon secondary antigen exposure, as demonstrated in studies comparing murine TCR transgenic cells with or without CTLA4 expression [5]. Data on a role of CTLA4 in limiting CD8^+^ T cell proliferation is more controversial [19], [20].

In addition, Ras signaling downstream of TCR/CD3 activates phosphoinositide 3-kinases (PI3K) leading to the phosphorylation of the serine/threonine kinases AKT, also known as protein kinases B (PKB), a pathway important for cell survival [21]. Given the antagonistic effects on TCR signaling, CTLA4 engagement would be expected to inhibit this pathway, which has been demonstrated by some groups [22], [23]. However, CTLA4 is also known to bind the phosphatases SHP-2 and PP2A, two inhibitors of AKT [14], [24]. In some experimental systems it has been shown that ligation of CTLA4 activates PI3K and AKT, resulting in inhibition of Bcl-2 apoptotic family members and increased cell survival [25]. The significance of AKT activation and apoptosis inhibition has been explained as CTLA4 inhibiting cell proliferation through the inhibition of MAPKs and at the same time promoting cell survival through the activation of PI3K/AKT, resulting in T cell anergy and tolerance without T cell death [25]. It is currently unknown if these opposing effects can be explained by different assay systems or by analyzing different immune cell subsets. Therefore, we reasoned that co-staining with surface molecules to identify immune cell subsets at a single cell level and to determine the effects on phosphorylated PI3K and AKT in PBMC obtained from patients receiving a CTLA4 blocking antibody, together with molecules involved in cell cycle (cyclin D1) and apoptosis (bcl-2), may provide information on the effects of CTLA4 blockade in humans.

The second network studied by us was signaling immediately downstream of cytokine receptors through signal transducers and activators of transcription (STAT). STATs are a family of cytoplasmic transcription factors that intercede the signaling by cytokines. Upon cytokine binding to their receptor, STATs are activated by Janus kinases (JAK) through phosphorylation of tyrosine residues resulting in dimerization and migration to the nucleus to regulate gene expression. Our primary intent was to determine if exposure to CTLA4 blockade resulted in altered responsiveness to cytokine activation, since CTLA4 has been long recognized to inhibit IL-2 transcription and to lower production of immune activating cytokines [3]. In addition, evidence has been provided for a potential direct interaction between CTLA4 and STAT5 using a two-hybrid yeast system [15], although the relevance of these findings to primary human T cells is currently unknown. Furthermore, certain STATs can be additionally phosphorylated by src protein kinases and MAPK kinases [26], [27], thereby being potential downstream effectors of TCR and costimulatory activation. Therefore, we anticipated that blockade of CTLA4 would result in a direct or indirect increase in phosphorylated STAT proteins.

Work pioneered by Garry Nolan’s group has provided a technique to allow the quantitative study of activated proteins within cell signaling networks through multiparameter flow cytometry, with simultaneous surface and intracellular staining [28]. This technique, termed phosphoflow, is based on the use of antibodies specific for intracellular phosphorylated proteins in permeabilized cells [28], [29]. Phosphoflow allows the study of cell signaling pathways at a single cell level, with the benefit of allowing a concomitant characterization of the cell type being studied using polychromatic surface flow cytometry staining. Aided by this technique, in the current work we explored the effects of the anti-CTLA4 antibody tremelimumab on the phosphorylated state of key effectors of the TCR and cytokine signaling pathways within immune subsets of cells obtained from peripheral blood of patients with metastatic melanoma. Our studies provide evidence of the selective effects of blocking CTLA4 in humans at the cellular and molecular level.

## Materials and Methods

### Clinical Trials and Study Samples

Peripheral blood samples were obtained after written informed consent from 27 patients with stage IIIc or IV melanoma treated at UCLA within two protocols based on the administration of tremelimumab (Pfizer, New London, CT). This research was approved by the UCLA Internal Review Board (IRB) with approval numbers of 03-12-023, 04-07-063 and 06-06-093. These approvals allowed the collection of PBMC after signing a written informed consent and their analyses using the immune monitoring assays described in this manuscript. Six patients were treated within a phase I clinical trial of three biweekly intradermal (i.d.) administrations of a fixed dose of 1×10^7^ autologous DC pulsed with the MART-1_26–35_ immunodominant peptide epitope (MART-1/DC) manufactured as previously described [30], concomitantly with the intravenous (i.v.) administration of a dose escalation of tremelimumab at 10 (3 patients) and 15 mg/kg (3 other patients) every 3 months (UCLA IRB# 03-12-023, IND# 11579, clinical trial registration number NCT0090896). Samples from these patients were coded with the study denomination of NRA and a patient-specific number. The remaining 21 patients were enrolled in a phase II clinical trial of single agent tremelimumab (UCLA IRB# 06-06-093, IND# 100453, clinical trial registration number NCT00471887) administered at 15 mg/kg every 3 months. Samples from these patients were coded with the study denomination of GA and a patient-specific number. Objective clinical responses were recorded following a modified Response Evaluation Criteria in Solid Tumors (RECIST) [31], where skin and subcutaneous lesions evaluable only by physical exam were considered measurable if adequately recorded using a photographic camera with a measuring tape or ruler; there was no minimum size restriction for these lesions. PBMC were obtained from two healthy subjects by leukapheresis under UCLA IRB#04-07-063 and discarded healthy donor PBMC were obtained from three anonymized samples from the virology core at UCLA. These PBMC were used to perform *in vitro* monocyte staining and anti-CTLA4 antibody titration experiments.

### Sample Procurement and Processing

PBMC were collected from patients receiving tremelimumab-containing experimental immunotherapy by leukapheresis as previously described [32]. Leukaphereses were planned as part of the pre-dosing procedures, and between one and two months after receiving the first dose of tremelimumab. Phosphoflow studies were run using aliquots of cryopreserved PBMC thawed and immediately diluted with RPMI complete media containing 5% human AB serum and 1% penicillin, streptomycin, and amphotericin (Omega Scientific, Tarzana, CA). Cells were washed and subjected to enzymatic treatment with DNase (Sigma, St. Louis, MO) for 1 hour at 37°C, and rested overnight in a 5% CO_2_ incubator.

### Optimization of the Cell Surface Staining Protocol

We first optimized the conditions and antibody clones for simultaneous cell surface and intracellular staining. We analyzed the performance of commercially-available antibodies for cell surface staining in fresh cells compared to cells fixed with 2% paraformaldehyde (Electron Microscopy Services, Fort Washington, PA) and permeabilized using 90% ice-cold methanol. The following antibodies were tested: phycoerytherin-Cy5 (PE-Cy5)-CD3 (UCHT1, Beckman Coulter, Fullerton, CA), allophycocyanin-Cy7 (APC-CY7)-CD4 (RPA-T4) and PE-CD8 (RPA-T8, BD Biosciences); Pacific blue (PacBlue)-CD14 (TüK4) and PacBlue-CD8 (clone 3B5, Invitrogen, Carlsbad, CA), PacBlue-CD8 (clone OKT8, eBioscience, San Diego). For all flow cytometry experiments, a combination of anti-mouse Igκ/Negative Control Compensation Particles (BD Biosciences) and PBMC were used for compensation purposes. The fluorescent minus one (FMO) approach was used to gate appropriately [32], [33]. Samples were acquired in a LSR II Flow Cytometer (BD Biosciences). All flow data analysis was done with the FlowJo software (Tree Star Inc., Asland, OR), and plotted with GraphPad Prism (GraphPad, San Diego, CA) for statistical analysis and graphing. We collected and depicted the data using bi-exponential axis.

### Cellular Barcoding, Surface Staining and Intracellular Phospho-specific Flow Cytometry

Paraformaldehyde-fixed PBMC were washed twice in 3 ml of phospho-staining buffer (Dulbecco phosphate buffer saline, DPBS), 0.5% bovine serum albumin (BSA), and 0.01% sodium azide. At the time of permeabilization with 90% ice-cold methanol, post-dosing samples were labeled following the cellular barcoding technique [34] with 5 and 6 µg/ml of Pacific orange-succinimidyl ester (-NHS) and Ax350-NHS (Invitrogen, Carlsbad, CA) respectively, and the pre-dosing samples were left unstained, resulting in two barcoded samples that were then combined into the same tube for both surface and intracellular staining. The intracellular staining antibodies were used at saturating conditions (see Table S1 for antibody clones, flurochrome labeling and vendors). PBMC were stored at −20°C for up to three days until flow cytometry analysis, as described [28], [29], [35].

### Cytokine Stimulation Experiments

We first optimized the protocol to define the cytokine concentration for maximal pSTAT detection using ten-point dose-response curves of titrated concentrations of several human cytokines in geometric series. The following cytokines were titrated: interleukin 2 (IL-2, gift from Novartis, Emeryville, CA), IL-4 (CellGenix, Freiburg, Germany); IL-6 and interferon-gamma (IFN-γ, BD Biosciences); IL-7 (eBioscience); IL-15 (gift from Amgen, Thousand Oaks, CA); and interferon-alpha (IFN-α, Chemicon/Millipore, Billerica, MA). Downstream TCR signaling experiments were performed using media containing 300 IU/ml of IL-2 and 50 ng/ml of OKT3 (eBioscience, San Diego,CA) at different time points. PBMC and cytokines were incubated at 37°C, 5% CO_2_ for 15 minutes, and TCR signaling experiments were done with a 30 minute incubation. PBMC were fixed in 2% paraformaldehyde, permeabilized in 90% ice-cold methanol and surface and intracellularly stained for flow cytometry as described above.

### Anti-CTLA4 Stimulation *In Vitro* Experiments

To obtain monocytes, thawed PBMC from one healthy subsject and three patients with metastatic melanoma were allowed to adhere after overnight incubation. For the detection of CTLA4 expression by monocytes, adherent PBMC were scraped and stained with anti-CD3, anti-CD14 and anti-CTLA4 for CTLA4 surface stainig, or surface stained with anti-CD3 and anti-CD14 before fixation and permeabilization with iTAg fixative and permeabilization reagents (Beckman Coulter), and then intracellularly stained with anti-CTLA4-PE antibody. All antibodies used in these studies are detailed in Table S2. For *in vitro* studies of anti-CTLA4 engagement in monocytes, tremelimumab was added to the adherent PBMC at titrated concentrations. After 2 days in culture, cells were fixed and permeabilized for phosphoflow analysis as described above. During the permeabilization time, samples underwent fluorescent cell barcoding [34]. Briefly, samples were labeled with a combination of 0, 3 or 8 µg/ml of Ax350-NHS and 0 or 3 µg/mlof Ax750-NHS, or a combination 0 or 6 µg/ml of Ax350-NHS and 0 or 3 µg/ml of Ax750-NHS. This approach allowed the simultaneous analysis of four to six different populations in the same sample. Barcoded monocyte samples were analyzed using a combination of surface and intracellular staining antibodies detailed in Table S2.

### Statistical Analysis

In all cases we collected one million CD3^+^ lymphocytes through flow cytometry. Mean fluorescent intensity (MFI) was used as a measure of pre- and post-treatment basal change. GraphPad Prism (GraphPad Software, San Diego, CA) package program and Microsoft Excel were used for data presentation. Two-tailed paired Student t-test was used to compare pre- and post-treatment tremelimumab effects.

## Results

### Patient Characteristics and Sample Procurement

Cells collected from peripheral blood by leukapheresis from 27 patients enrolled in two clinical trials administering tremelimumab were analyzed (Table 1). Six patients received tremelimumab together with MART-1_26–35_/DC (NRA study) and 21 patients received tremelimumab alone (GA study). The majority of patients had M1c metastatic melanoma (visceral metastasis and/or high LDH). Overall, 6 patients (4 in the GA study, 2 in the NRA study) had an objective tumor response, resulting in sustained and durable tumor regressions in 5 of them. These 6 patients had either stage IIIc or M1a metastatic melanoma. Major toxicities included two cases of grade 3 diarrhea or colitis and one case of symptomatic panhypopituitarism (grade 2 hypophysitis). None of these patients received corticosteroids or other immune suppressive treatment before the post-dosing sample collection for the phosphoflow analysis. Due to differences in clinical trial design the post-dosing leukaphereses were performed at a median of 38 days after the dose of tremelimumab in the GA cohort, and at a median of 71 days in the NRA cohort. In both cases, serum levels of tremelimumab were expected to be above 10 µg/ml at the time of blood cell collection, a concentration of this antibody consistent with CTLA4 blockade *in vitro* [9], [10].

**Table 1:** Patient characteristics.

		Patient cohort	
		NRA	GA
Number of patients		6	21
Mean age (range)		56 (34–61)	52 (27–81)
Female/male		2/4	4/17
Stage	IIIc	1	3
	M1a	2	2
	M1b	0	2
	M1c	3	14
Prior therapy	Surgery only	1	13
	Immunotherapy only	3	3
	Chemotherapy only	1	3
	Immunotherapy and chemotherapy	1	2
Tremelimumab dose	10 mg/kg q3 months	3	0
	15 mg/kg q3 months	3	21
MART-1/DC		6	0
Toxicities	Grade 2 pruritus	0	2
	Grade 2 diarrhea	0	3
	Grade 2 hypophysitis	0	1
	Grade 3 colitis	0	2
Response	Partial response	1	1
	Complete response	1	3

### Phosphoflow Technique Optimization

We first established an optimized combination of antibodies against cell surface and intracellular signaling molecules. Cell permeabilization with the use of methanol had a significant influence on the quality of both surface and intracellular staining. The expression of CD8 after cell permeabilization was detected with only one of three antibodies tested (Figure S1). Additionally, in preparation for cytokine stimulation studies, different cytokines at increasing concentrations were tested to generate a sufficient dose-response curve to obtain the highest phosphorylation signal after 15 minutes of cytokine stimulation. The optimal cytokine concentrations were obtained following a geometric series determining a plateau between cytokine concentration and maximum specific phosphorylated protein detection (see Figure S2 and Table S3 for optimization of the time-course for TCR stimulation experiments).

### Simultaneous Analysis of Pre- and Post-dosing Samples with Combined Surface and Intracellular Staining

**Figure 1:**
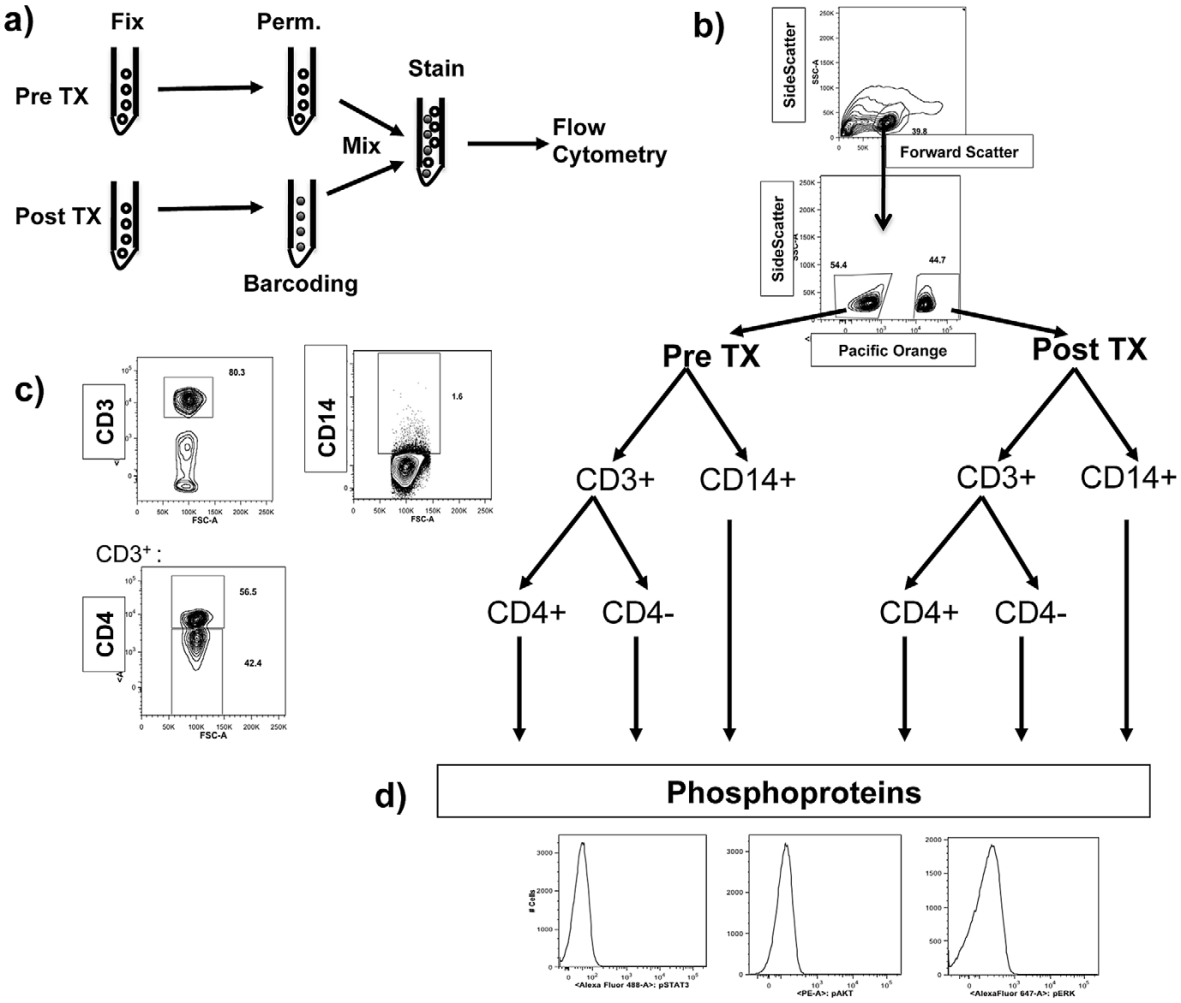
Outline of flow cytometry staining, cellular barcoding and phosphoflow analysis. a) Fixation, permeabilization, fluorescent barcoding and surface and intracellular staining sequence for pre- and post-tremelimumab treatment samples. b) Gating pyramid. After gating by morphology, pre- and post-dosing samples were resolved based on the Pacific orange barcoding cell labeling. c) The cells from pre- (negative for Pacific orange) and post-treatment (positive for Pacific orange) time points were then separated as CD3^+^ (T lymphocytes) and CD14^+^ (monocytes). The CD3^+^ cells were gated on CD4^+^ to analyze T helper cells, and CD4^−^ cells to analyze for CD8^+^ cytotoxic T lymphocytes (CTLs). d) Intracellular phosphoproteins were analyzed in each cell subset by mean fluorescence intensity (MFI).

We wanted to simultaneously analyze pre- and post-dosing PBMC samples to minimize within-run analytical variability while allowing detailed analysis for each major immune cell subset. Therefore, a two cellular-barcoded approach was used in which the post-dosing samples were labeled with Pacific orange-NHS and combined with unlabeled predosing-samples, and both populations were stained for the expression of surface and intracellular molecules (Figure 1a). First, side and forward scatters were used to determine cellular morphology followed by resolution of the cellular barcoding into the two original populations (pre- and post-dosing samples, Figure 1b). Then, the identification of immune cell subsets was performed using a combination of CD3, CD4 and CD14, where CD4^+^ T helper cells were detected as double positive CD3^+^CD4^+^ cells, CD8^+^ CTLs as CD3^+^CD4^−^ and/or CD3^+^CD8^+^, and monocytes as CD3^−^CD14^+^ (Figure 1c). The MFI of each phosphoprotein was measured for each individual cell subset (Figure 1d).

### Modulation of TCR and Cytokine Receptor Signaling After Administration of Tremelimumab Together with Dendritic Cell Vaccines

**Figure 2:**
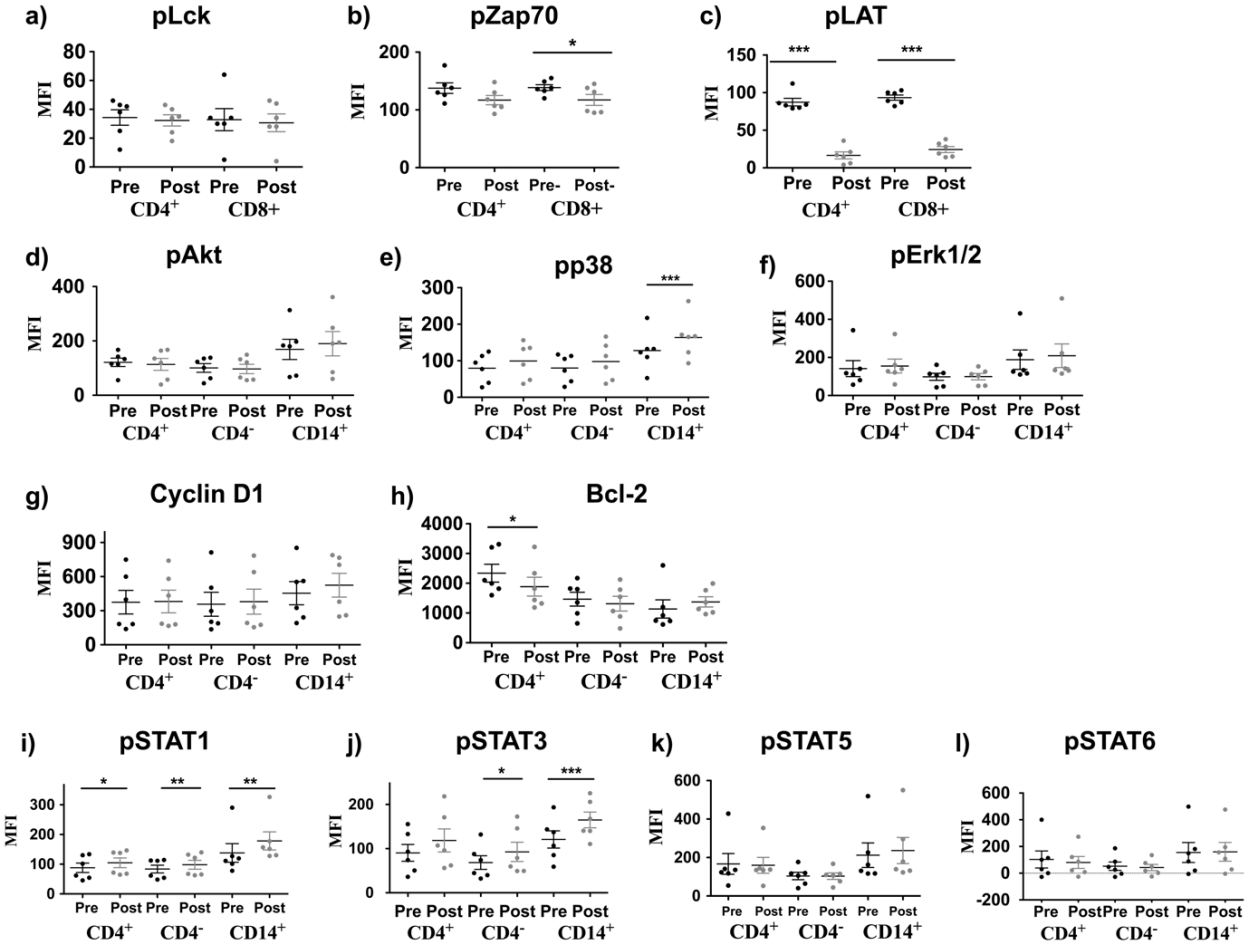
Basal levels of TCR and cytokine signaling pathways in samples taken from patients in the NRA study of tremelimumab administered together with MART-1_26–35_ peptide pulsed dendritic cells (DC). The MFI of phosphoproteins was measured in CD3^+^CD4^+^ (T helper), CD3^+^CD4^−^ (mainly representing CD8^+^ CTLs) and CD3^−^CD14^+^ (monocyte) cell subsets. PBMC were obtained from patients before (Pre) and after (Post) receiving the combined therapy. a) pLck (pY505), b) pZAP70 (pY292) and c) pLAT (pY171) representative of proximal T cell receptor (TCR) activation, which was only analyzed in CD3 gates including CD4 and CD8 cells. d) pAKT (pT308), e) p38 (pT180/pY182), f) pERK1/2 (T202/204), correspond to the main intracellular signaling pathways downstream of surface receptors, including the TCR. g) Cyclin D1 and h) bcl2 were measured as whole protein content for surrogate evidence for modulation of the cell cycle and apoptosis, respectively. i) Phospho-STAT1 (pY701), j) pSTAT3 (pY705), k) pSTAT5 (Y694) and l) pSTAT6 (Y641) were studied as a measure of STAT signaling downstream of cytokine receptors. In all cases, p values were calculated using a two-sided paired t-test, and significant results are denoted with a line comparing pre- and post-dosing samples with an asterisk to represent the significance level as follows: *p < 0.05; **p < 0.01; ***p < 0.001. Y-axis =  MFI - mean fluorescent intensity. Each dot represents the average of six replicates in most instances, and twenty-four replicates for pSTAT 1 and pSTAT5. The horizontal bar in each scatter column indicates medians. The vertical bar in each scatter column indicates standard error.

We first analyzed changes in phosphoprotein expression in post-dosing samples from 6 patients treated within the NRA study (combination of tremelimumab and MART-1_26–35_/DC vaccines, Figure 2). In the post-dosing population, phosphorylation of the proximal src kinases pLAT (p<0.001) and pZAP70 (p<0.05) decreased in CD8^+^ (Figure 2b, c) but no changes were detected in pLck or pAKT (Figure 2a, d). There was a significant increase in pp38 in CD14^+^ monocytes (p<0.001), with a similar but non-significant trend in both T cell subpopulations (Figure 2e). Analysis of whole protein levels of cyclin D1 (to explore cell cycle changes, Figure 2g) and bcl-2 (to explore apoptotic molecule changes, Figure 2h) did not demonstrate significant changes, other than a small significant post-dosing decrease in bcl-2 protein only in CD4^+^ cells (p<0.05). Post-dosing levels of pSTAT1 and pSTAT3 increased consistently in all cell subsets, being statistically significant in all cases except for pSTAT3 in CD4^+^ cells (p<0.05, Figure 2i, j). Post-dosing pSTAT5 and pSTAT6 were not significantly different from pre-dosing values (Figure 2k, l).

### T Cells and Monocytes Functionally Respond to Cytokine Stimulation Without Differences Between Pre-dosing and Post-dosing Samples

We then analyzed if *in vivo* exposure to tremelimumab would enhance the ability of cells to respond to cytokine pulsing by phosphorylating STAT proteins. IFN-α is known to signal through STAT1 and STAT5, and pulsing with this cytokine led to a consistent increase in pSTAT1 as expected (Figure S3). However, there were no differences in pSTAT1 and pSTAT5 levels after stimulation with IFN-γ (data not shown). We also analyzed pSTAT5 after pulsing with the common gamma-chain (γc) signaling Th1 cytokines IL-2, IL-7 and IL-15. pSTAT5 increased after cytokine stimulation, but there were no detectable changes comparing pre- and post-tremelimumab treatment samples (Figure S4). When stimulating cells with the Th2 cytokine IL-4, which also signals through the common γc receptor, there were no differences in pSTAT1 and pSTAT5, but there was a significant increase in pSTAT6 in all three cell subsets. As with the other cytokines, there were no differences in stimulated pre- and post-tremelimumab treatment samples (data not shown). Taken together, treatment with tremelimumab and MART-1_26–35_/DC vaccines did not alter the ability of T cells or monocytes to respond to cytokine stimulation.

### Detection of CTLA4 and Signaling Changes Upon CTLA4 Blockade in Monocytes

**Figure 3:**
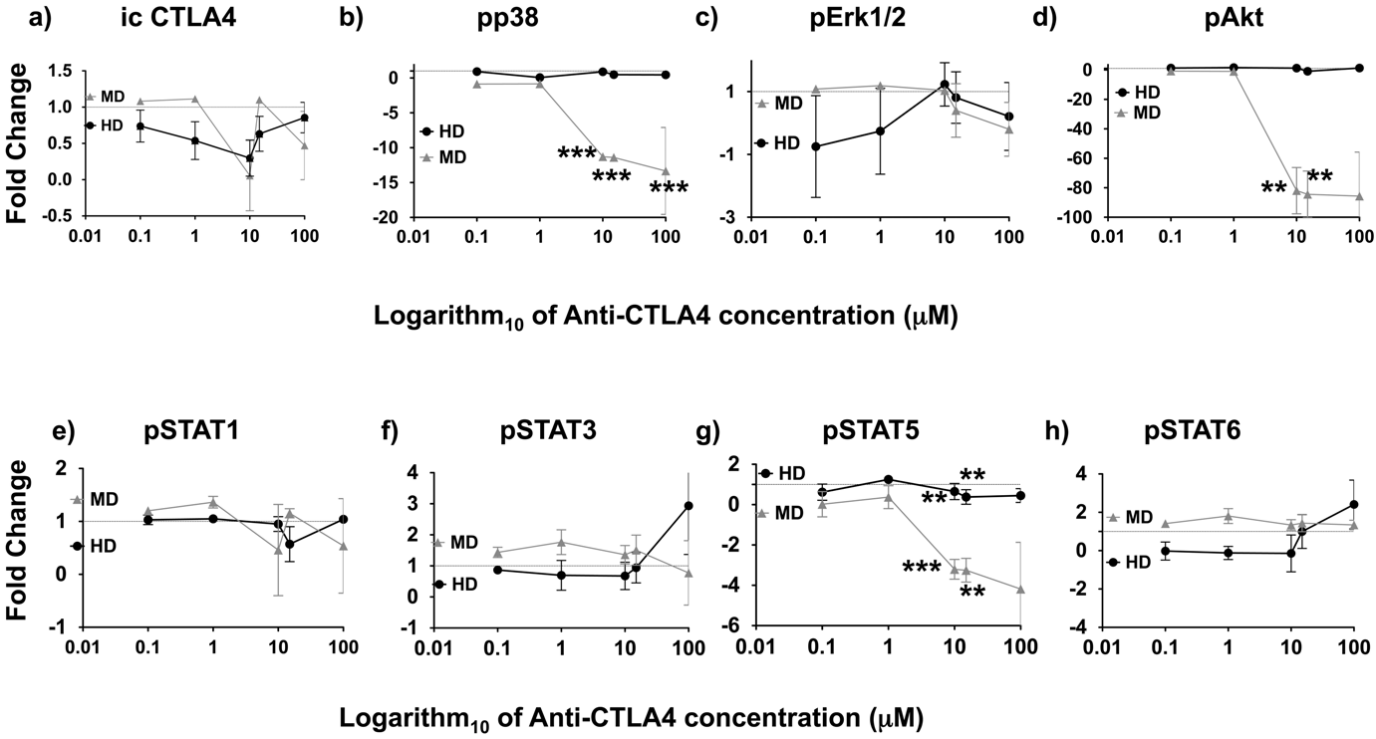
Modulation of CTLA4 and phosphoprotein expression in monocytes exposed *ex vivo* to tremelimumab. Monocytes were cultured in increasing concentrations of tremelimumab for 48 hours, after which were analyzed for intracellular flow cytometry. Data is presented as fold change from the baseline mean fluorescence intensity (MFI) at increasing concentrations of tremelimumab (0, 0.1, 1. 10. 15 and 100 µg/ml) presented in a semilogarithmic plot. Red triangles represent monocytes samples obtained from three patients with metastatic melanoma (MD: melanoma donors); black circles are results from monocytes analyzed from five healthy subjects (HD: healthy donors). a) Intracellular CTLA4 expression; b) pp38(pT180/pY182); c) pErk1/2 (T202/204); d) pAkt (pT308); e) pSTAT1(pY701); f) pSTAT3 (pY705); g) pSTAT5 (Y694); h) pSTAT6 (Y641). **p < 0.01; ***p < 0.001.

After repeatedly detecting changes in signaling phosphoproteins in CD14^+^ monocytes obtained from patients after treatment with tremelimumab, we performed *in vitro* experiments with monocytes from a healthy subject and three patients with metastatic to confirm the expression of CTLA4 and the modulation of phosphoproteins upon CTLA4 blockade. There was no significant detection of surface CTLA4 on monocytes by flow cytometry (0.10±0.02%, 13 replicates, n = 8 subjects) but we could readily detect intracellular CTLA4 in monocytes (89.93±1.40%, 30 replicates, n = 8 subjects), with no difference in either staining between a healthy subject and patients with melanoma (a representative example of surface and intracellular staining for CTLA4 in monocytes is included in Figure S5a). After a 48-hour incubation with tremelimumab *in vitro* the intracellular expression of CTLA4 in monocytes decreased, demonstrating target modulation even with undetectable surface expression of CTLA4 (Figure 3). Phosphorylation of Erk, STAT1, STAT3 and STAT6 did not change significantly with *ex vivo* tremelimumab exposure in samples from five healthy donors and three patients with metastatic melanoma, while intracellular pp38, and pAKT decreased significantly from baseline with increasing concentrations of tremelimumab only in samples from patients with melanoma (Figure 3 and Figure S5). Phosphorylation of STAT5 decreased significantly with increasing concentrations of tremelimumab in monocytes from five healthy donors and from three patients with melanoma. Overall, these data demonstrate that monocytes express mostly intracellular CTLA4 and that CTLA4 is biologically active in this cell subset since exposure to tremelimumab induces changes in intracellular signaling molecules.

### Confirmation of the Modulation of TCR and Cytokine Receptor Signaling After Administration of Tremelimumab

**Figure 4:**
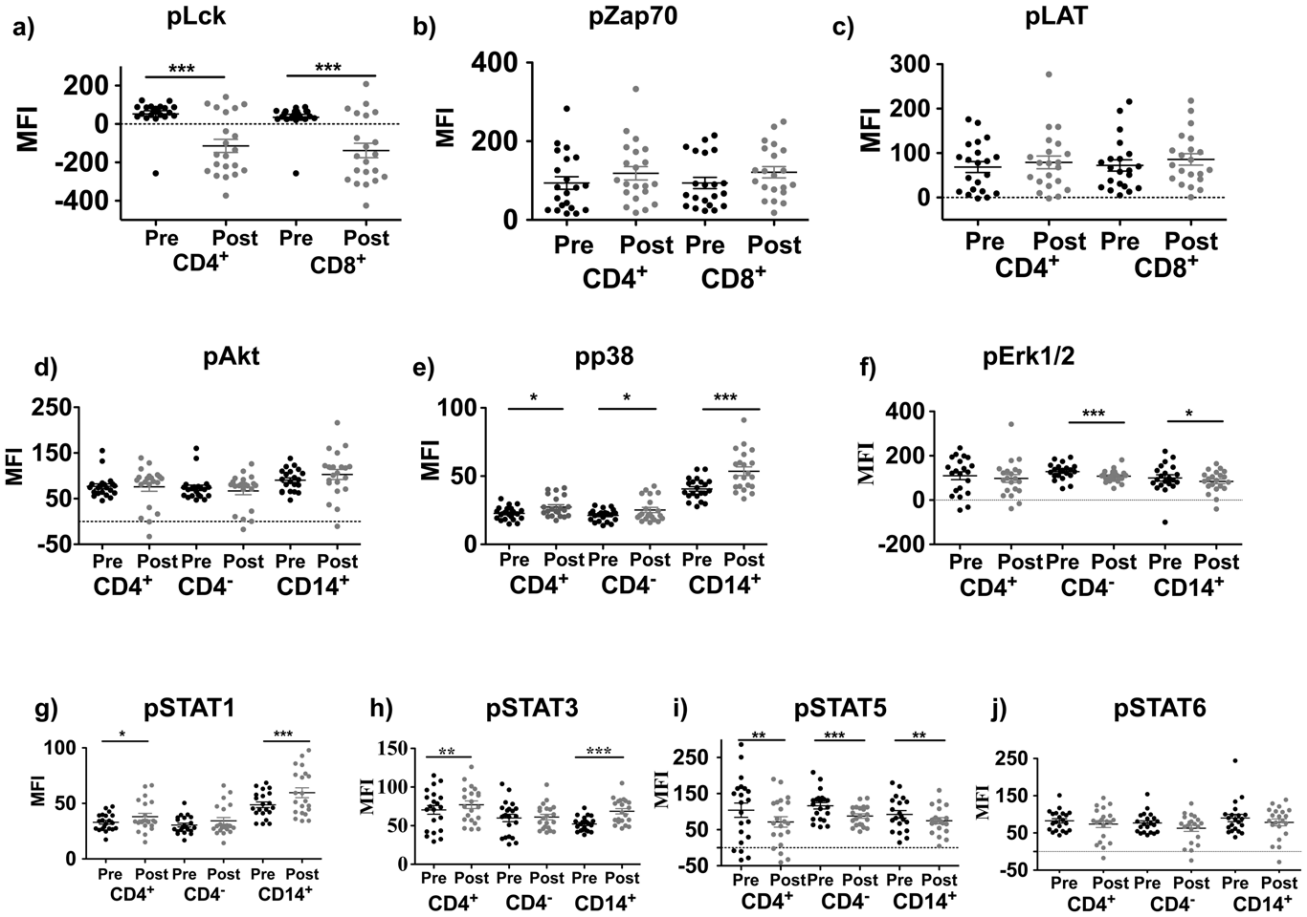
Basal levels of TCR and cytokine signaling pathway activation in samples taken from patients in the GA study administering single agent tremelimumab. PBMC obtained from patients before (Pre) and after (Post) single agent tremelimumab were analyzed by phosphoflow as described in Figure 2 within each of the three cell subsets: CD4^+^, CD8^+^ (gated as CD3^+^CD4^−^) and CD14^+^ cells. a) pLck (pY505), b) pZAP70 (pY292), and c) pLAT (pY171) representative of proximal T Cell receptor (TCR) activation, only analyzed in CD3 subsets. d) pAKT (pT308), e) pp38 (pT180/pY182) and f) pERK1/2 (T202/204) as intracellular signaling pathways downstream of surface receptors. g) Phospho-STAT1 (pY701), h) pSTA3 (pY705), i) pSTAT5 (Y694), and j) pSTAT6 (Y641) as a measure of cytokine receptor signaling. Statistically significant results are denoted with asterisks as described in Figure 2.

Our most consistent effects in post-tremelimumab treatment samples from the NRA study were changes in pp38, pLAT, pZAP70, pSTAT1 and pSTAT3 in the absence of additional *ex vivo* TCR or cytokine stimulation (Figure 2). These results were based on the analysis of paired samples from only 6 patients, and this study had the confounding effect of the administration of a MART-1_26–35_ peptide pulsed DC vaccine in addition to tremelimumab. Therefore, we decided to confirm these changes in a validation set of samples taken from the GA study using paired samples from 21 patients who received single agent tremelimumab at 15 mg/kg every 3 months (Figure 4). In this larger dataset we confirmed a statistically significant increase in pp38 for the three cell subsets (CD4, CD8, CD14) in post-dosing samples. Surprisingly, we detected decreases in pLck (statistically significant in all cell subsets; p<0.001), and pERK1/2 (significant in CD8+ and CD14+ cells, p<0.05), but no significant changes in pLat, pZap70 or pAkt. Analysis of phosphorylated STAT proteins (without additional *ex vivo* cytokine stimulation) confirmed the increasing trend in pSTAT1 and pSTAT3 in post-dosing samples, in both cases statistically significant in the CD4^+^ (p<0.05) and CD14^+^ subsets (p<0.001). In addition, we also noted a consistent decrease in pSTAT5 in post-dosing samples, which was statistically significant in all three cell subsets (p<0.01).

### Differential Phosphorylation Patterns in Patients with Clinical Response to Tremelimumab

**Figure 5:**
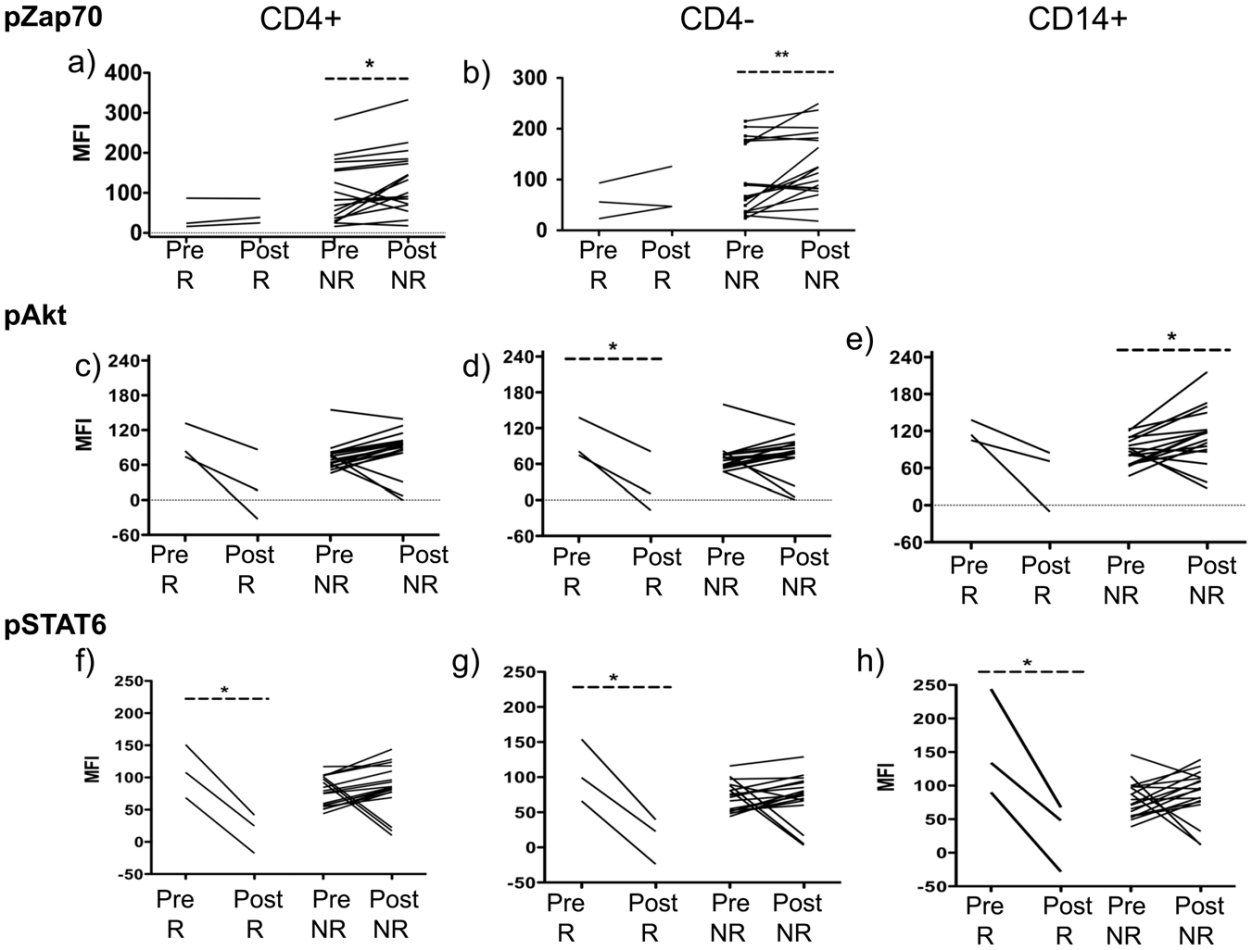
Analysis of signaling patterns in samples from clinically responding and non-responding patients in the GA study administering single agent tremelimumab. Phosphoflow results from patients before (Pre) and after (Post) single agent tremelimumab separated among patients with a clinical response (R) or no response (NR). Samples were analyzed by phosphoflow as described in Figure 2. a and b) pZAP70 (pY292) in CD4^+^ and CD8^+^+ cell subsets. c, d and e) pAkt (pT308) in CD4^+^+, CD8^+^ and CD14^+^ cell subsets. f, g, and h) pSTAT6 (Y641) in all three cell subsets. Statistically significant results are denoted with asterisks as described in Figure 2.

Finally, we explored if samples from patients with an objective response to therapy had different patterns of changes in the signaling pathways compared to patients without objective tumor responses. This was done in the GA larger dataset, comparing three patients with a durable complete response with 18 patients with disease progression. Of note, there were no significant differences in the mean time of the post-dosing leukapheresis between patients with or without a clinical response. The general trends of decrease in pLck, pErk1/2 and pSTAT5 and increase in pp38, pSTAT1 and pSTAT3 continued to be evident both among clinical responders and non-responders when analyzed in two separate subgroups (data not shown). However, there were divergent trends between these two groups of patients in terms of pZap70, pAkt and pSTAT6 (Figure 5). Patients with a complete clinical response to therapy had statistically significant decreases in post-treatment pSTAT6 in all three cell subsets (p<0.05), while patients without a clinical response had minimal changes or even a trend towards increase in these two phosphorylated molecules (Figure 5a to c). The same trend was evident for pAkt, with the decrease in clinical responders only statistically significant in CD8 cells (p<0.05, Figure 5b). All three cell subsets showed an increasing trend in pAkt in post-dosing in samples from the clinical non-responders, which was significant for CD14^+^ cells (p<0.05). The proximal pZap70 showed a significant increase in CD8 cells (p<0.01) in non-responders. Therefore, this exploratory analysis suggests that patients with a clinical response to tremelimumab may display different immune cell activation patterns in peripheral blood.

## Discussion

Enabled by the phosphoflow technique, which permitted the concomitant multiparameter evaluation of surface and intracellular signaling patterns at a single cell level, we explored how the anti-CTLA4 antibody tremelimumab altered cell signaling pathways downstream of the TCR and cytokine receptors in peripheral blood cells obtained from patients with metastatic melanoma treated within two clinical trials. Phosphorylation was used as readout for either a constitutive or stimulus-induced pathway activation, thereby providing both phenotypic and functional information [29]. Our data analysis unraveled that tremelimumab-based therapy has a preferential effect on intracellular signaling pathways mainly in CD4^+^ and CD14^+^ cells. CD4^+^ T cell activation by anti-CTLA4 antibodies, detected as increase in the surface marker HLA-DR, was the earliest described activation effect of these antibodies on the human immune system [32], [36], [37], which has been expanded to the detection of activation-induced ICOS surface expression upon CTLA4 blockade [38], [39]. However, there has not been much preclinical or clinical data supporting an increased activation state of monocytes, even though this cell subset has been previously shown to expresses CTLA4 [8]. We noted a consistent increase in phosphorylated p38, STAT1 and STAT3 in monocytes, with most of these changes being highly significant in samples from both clinical trials with tremelimumab. In our studies we confirmed the expression of CTLA4 in monocytes, which was mostly intracellular, with weak direct modulating effects of tremelimumab on signaling phosphoprotein networks.

The most solid findings derived from the initial analysis of the NRA combined therapy study were confirmed in the GA single agent study, but results differed slightly. Differences in results between both studies can be explained by several reasons. The addition of MART-1_26–35_/DC vaccines in the NRA study, which was aimed at expanding CD8^+^ T cells specific for the MART-1 melanoma antigen, is unlikely to have a major impact since the population of MART-1-specific T cells expanded by the DC vaccine is very small [30], [40]. Another possibility is the difference in dose of tremelimumab as three patients in the NRA combination study received a lower dose of tremelimumab at 10 mg/kg. However, we did not detect evidence of tremelimumab dose-response effects in these studies. Another source of variability is the different timing of leukapheresis, with a median of over 2 months from the first dose of tremelimumab in the NRA study compared to approximately one month in the GA study. However, pharmacokinetic analysis predict that serum tremelimumab concentrations should be above 10 µg/ml up to three months both in the 10 mg/kg and 15 mg/kg doses [10], which is the minimum concentration of this antibody correlating with a biological effect [9]. The fact that most results are evident and concordant in both series, but more likely to be significant in the larger GA sample set, turns us to believe that the difference in the number of patients (6 compared to 21) may be the major factor responsible for the differences in statistically significant results.

Contrary to our expectations, we noted a consistent decrease in the phosphorylation of the early TCR signaling molecules Lat, Lck and Zap70 in the post-dosing samples of patients receiving therapeutic CTLA4 blockade. Zap70 is a src kinases with a major role in proximal TCR signaling, and it would have been expected that upon CTLA4 blockade there should be enhanced signaling from the TCR. In preclinical models it has been well established that engagement of CTLA4 leads to decreased phosphorylation of Lck and Zap70 [3], while CTLA4 knock out mice [14] and humans with polymorphisms in CTLA4 that lead to decreased inhibitory function [17], have constitutively increased phosphorylation in Lck and Zap70. In addition, the GA series demonstrated significantly decreased phosphorylation of Erk1/2 in post-dosing samples, a finding that also goes against our expectations based on data from preclinical models [18]. On the contrary, there was an increase in phosphorylated p38, also a MAPK signaling molecule. Collectively, these data suggest a decrease in most signaling molecules downstream of the TCR after continuous CTLA4 blockade in patients. An explanation would be that the time point of sampling (between 1 and 2.5 months after administration of tremelimumab in most cases) would detect late negative feedback loops triggered by continuous pathway activation that lead to inhibition of upstream phosphorylation or the activation of phosphatases [14], while activation of p38 would continue to be evident.

There was a consistent modulation of STAT signaling after the administration of tremelimumab. In all cell subsets and in both series there was evidence of increase in pSTAT1 and pSTAT3, with decrease in pSTAT5 and a non-significant but evident trend of pSTAT6 decrease. The STAT pathway is not the first one that comes to mind when thinking about downstream modulation after CTLA4 blockade. However, given the known effects of CTLA4 in altering cytokine production, in particular IL-2, and reports of STATs being phosphorylated downstream of the MAPK and Pi3K/Akt pathways [26], [27], [41], [42], it is not surprising that continuous antibody-mediated CTLA4 blockade would alter cytokine levels and lead to changes in the signaling through STATs [43]. In addition to the basal level analysis of STAT phosphorylation, we stimulated pre- and post-dosing PBMC with cytokines to determine if pre-dosing samples would be anergic and post-tremelimumab treatment samples would have increased sensitivity to cytokine stimulation. Our results demonstrated that pre-treatment samples have the same ability as post-treatment samples to signal upon cytokine stimulation. It has been previously reported that PBMC from patients with melanoma have decreased ability to respond to IFNs with an increase in pSTAT1 compared to healthy controls [44]. The absence of a healthy donor control group in our studies precluded us from being able to corroborate these observations.

A major finding in our studies is the unexpected profound alteration of signaling pathways in monocytes from patients treated with tremelimumab. In fact, post-dosing increases in phosphorylated p38, STAT1 and STAT3 in monocytes were the most consistent findings when comparing results from both datasets. These results point us to speculate that monocytes, a cell subset with known CTLA4 expression [8], may be a major and previously unrecognized player in the antitumor activity of anti-CTLA4-based therapy. We confirmed that monocytes do express CTLA4 but this is mostly intracellular. Direct engagement of CTLA4 on monocytes upon increasing concentrations of tremelimumab *in vitro* yielded minor changes on signaling phosphoproteins in monocytes, some of which were congruent with the findings in monocytes obtained from peripheral blood of patients treated with tremelimumab. This suggests to us that some of the effects we detected in monocytes may be a combination of direct modulation of CTLA4 on monocytes as well as indirectly due to changes in cytokine levels upon CTLA4 blockade in neighboring T cells. Interestingly, anti-TNF-α-based treatments are recognized as the most powerful immune suppressive treatment for the reversal of autoimmune toxicities induced by CTLA4 antibodies [45], [46]. Since monocytes are high producers of TNF-α, our observation of modulation of monocyte intracellular signaling may provide an explanation for the clinical activity of agents like infliximab.

In the exploratory analysis comparing peripheral blood samples from patients with and without a clinical response, we noted that three signaling molecules had a different pattern of change in samples from clinical responders. It is rather surprising to us that these changes were a decreased phosphorylation of the proximal TCR signaling molecules Zap70 and LAT, with concordant decrease in pAkt and pSTAT6. Samples from non-responding patients had either no change in the phosphorylation of these same signaling molecules, or an opposing increasing trend. The significance of these changes is unclear at this time and will require further validation in larger sets of patients with larger numbers of clinical responders. However, it is of interest to note that STAT6 has been described as a signaling molecule downstream of Akt [41], [42], suggesting to us that these changes after receiving tremelimumab may be reflective of a specific pathway modulation leading to more effective tumor targeting and killing.

In conclusion, tremelimumab-based therapy in patients with melanoma results in alterations of signaling pathways downstream of the TCR and cytokine receptors. This work provides a clear evidence of pharmacodynamic effects consistent with the suspected mechanism of action of CTLA4 blocking antibodies. Modulation of p38 and STATs was most prominent in CD14^+^ monocytes, suggesting that further studies focusing on the contribution of monocytes and differentiated macrophages or myeloid DCs may shed more light on the mechanism of action of CTLA4 blocking monoclonal antibodies. In addition, studying patterns of different phosphorylated signaling molecules in patients with or without clinical response may allow defining if clinical benefit is guided by a different modulation of the immune system by CTLA4 blocking antibodies.

## Supporting Information

Table S1 *Beckman Coulter and Invitrogen**, eBioscience***; the other antibodies from BD Biosciences; Ax =  AlexaFluor; PE =  Phycoerythrin; APC =  Allophycocyanin; Cy =  cyanine; PE-pSTAT6 (pY641; clone 18).
(0.04 MB DOC)

Table S2 *Beckman Coulter and Invitrogen**; the other antibodies from BD Biosciences; Ax =  AlexaFluor; PE =  Phycoerythrin; APC =  Allophycocyanin; Cy =  cyanine. All of the antibodies were the clones described in Table S1.
(0.04 MB DOC)

Table S3 Time course for activation TCR signaling in fold change from baseline.
(0.04 MB DOC)

Figure S1 Effects of cell fixation and permeabilization on the detection of surface staining for immune cell markers. Flow cytometric identification of CD3 (UCHT1), CD4 (RPA-T4), CD14 (TüK4), and CD8 (OKT8) populations by antibody staining in fixed and permeabilized human PBMC. PBMC from patients with advanced melanoma were kept alive or fixed with 2% paraformaldehyde (PFA) and permeabilized with 90% methanol (MeOH) before staining with the respective antibodies. CD8 (RPA-T8) staining could not be identified by flow cytometry after 2% paraformaldehyde/90% methanol treatment.
(3.82 MB TIF)

Figure S2 Titration of cytokines in healthy donor PBMC. a) Formula for the calculation of the geometric series. Max: maximum value of the curve, a =  min: minimum value of the curve, n: numbers of points in the curve. b) Dose-response curve for IFN- alpha [readout was pSTAT1 (pY701) and pSTAT5 (pY694)]; IFN-gamma [readout pSTAT1(pY701)]; IL-2, IL-7 and IL-15 [pSTAT5 (pY694)]; IL-6 [pSTAT3 (pY705)]; IL-4 [pSTAT6(pY641)]. c) Final concentration of saturating levels of cytokines used in this study were selected from the plateau of the curve.
(1.81 MB TIF)

Figure S3 Effects of IFN-alpha cytokine stimulation on phosphorylated STAT1 and STAT5 in samples taken from patients in the combined therapy NRA study. PBMC obtained from patients before (Pre) and after (Post) receiving tremelimumab together with MART-126-35 peptide pulsed dendritic cells (DC) were stimulated with 10,000 IU/ml of IFN-alpha for 15 minutes (S) and compared to unstimulated samples (U). Labeled cells were analyzed by phosphoflow for pSTAT1 (pY701, top row) or pSTAT5 (Y694, bottom row) as described in Figure 2. a and d) CD4+ CD3+ T helper cells. b and e) CD4-CD3+ cytotoxic T lymphocytes (CTLs). c and f) CD14+ monocytes. Statistically significant results are denoted with asterisks as described in Figure 2.
(0.74 MB TIF)

Figure S4 Effects of gamma chain receptor-mediated cytokine stimulation with IL-2, IL-7 and IL-15 on phosphorylated STAT5 in samples taken from patients in the combined therapy NRA study. PBMC obtained from patients before (Pre) and after (Post) tremelimumab together with MART-126-35 peptide pulsed dendritic cells (DC) were stimulated with 400 IU/ml of IL-2 (top row), 20 ng/ml of IL-7 (middle row) or 50 ng/ml of IL-15 (bottom row) for 15 minutes (S), and compared to unstimulated samples (U). Labeled cells were analyzed by phosphoflow for pSTAT5 (Y694) as described in Figure 2. a, d and g) CD4+CD3+ T helper cells. b, e and h) CD4-CD3+ cytotoxic T lymphocytes (CTLs). c, f and i) CD14+ monocytes. Statistically significant results are denoted with asterisks as described in Figure 2.
(2.22 MB TIF)

Figure S5 *In vitro* monocyte experiments. a) Representative results of extracellular and intracellular CTLA4 expression in monocytes. After gating monocytes by morphology, single cells were gated as CD14+CD3-. b) Gating strategy for phosphorylation experiments. After gating on monocytes by morphology, single cells were gated as CD14+CD3-. Six populations of cells, each one exposed to a different concentration of tremelimumab for 48 hours, were analyzed simultaneously based on fluorescent dye barcoding with Ax350-NHS and Ax-750-NHS. Each one of the differently labeled groups represents cells treated with increasing concentrations of tremelimumab in which intracellular phosphoproteins were analyzed by mean fluorescence intensity (MFI).
(2.32 MB TIF)
