# Supplementary material for: Modulation of Cell Signaling Networks after CTLA4 Blockade in Patients with Metastatic Melanoma
Source: PLoS One. 2010 Sep 15;5(9):e12711. doi: 10.1371/journal.pone.0012711 (PMC2939876; doi:10.1371/journal.pone.0012711)
Supplement: Table S1 — *Beckman Coulter and Invitrogen**, eBioscience***; the other antibodies from BD Biosciences; Ax = AlexaFluor; PE = Phycoerythrin; APC = Allophycocyanin; Cy = cyanine; PE-pSTAT6 (pY641; clone 18). (0.04 MB DOC) [file pone.0012711.s001.doc]

**Supplemental Table 1:** Antibody combinations for combined surface immunophenotyping and intracellular phosphoprotein analysis. In parenthesis the clone used.

| **Fluorochrome** | **Antibody** | | | | | | | | |
| --- | --- | --- | --- | --- | --- | --- | --- | --- | --- |
|  | **Cocktail 1** | **Cocktail 2** | **Cocktail 3** | **Cocktail 4** | **Cocktail 5** | | **Cocktail 6** | **Cocktail 7** | |
| PE-Cy5 | CD3 (UCHT1)* | | | | | | | | |
| APC-Cy7 | CD4 (RPA-T4)** | | | | | | | | |
| PacBlue | CD14 (TüK4) | | | | | | | | CD8 (OKT8) |
| Ax488 | pSTAT1- pY701  (4a) | CyD1 (G124-326) | pSTAT1-pY701 | pSTAT3-pY705 (4/P-STAT3), | | pSTAT3-pY705 | pP38 -pY180/pY182 (36/p38) | | pLAT-pY 171 ( I58-1169),) |
| sPE | pSTAT6-pY641 (18) | Bcl 2  (Bcl-2/100) | - | pSTAT1-pY701 | | pAKT-pT308  (J1-223.371) | - | | pZAP70-pY292  (J34-602) |
| Ax647 | pSTAT5-pY694  (47) | pERK1/2-pT202/Y204  (20a) | pSTAT6-pY641 ( 18), | pSTAT5-pY694 | | pERK1/2-pT202/Y204 | pSTAT5-pY694 | | pLck-pY505  (4/LCK-Y505) |
| Cytokine:  Pulsed | IFNγ | IFNγ | IL4 | IL6 | | IFNα | IL2/IL7/IL15 | | OKT3/IL2 |
