# Supplementary material for: Modulation of Cell Signaling Networks after CTLA4 Blockade in Patients with Metastatic Melanoma
Source: PLoS One. 2010 Sep 15;5(9):e12711. doi: 10.1371/journal.pone.0012711 (PMC2939876; doi:10.1371/journal.pone.0012711)
Supplement: Table S2 — *Beckman Coulter and Invitrogen**; the other antibodies from BD Biosciences; Ax = AlexaFluor; PE = Phycoerythrin; APC = Allophycocyanin; Cy = cyanine. All of the antibodies were the clones described in Table S1. (0.04 MB DOC) [file pone.0012711.s002.doc]

**Supplemental Table 2:** Antibody combinations for combined surface immunophenotyping and intracellular phosphoprotein analysis.

| **Fluorochrome** | **Antibody** | | |
| --- | --- | --- | --- |
|  | **Cocktail 1** | **Cocktail 2** | **Cocktail 3** |
| PE-Cy5 | CD3 * | | |
| PacBlue | CD14 ** | | |
| Ax488 | pSTAT1 | pSTAT3 | pSTAT6 |
| sPE | Anti-CTLA4 (BNI3) | Anti-CTLA4 | pAKT |
| Ax647 | pSTAT5 | pP38 | pERK1/2- |
