# Supplementary material for: Modulation of Cell Signaling Networks after CTLA4 Blockade in Patients with Metastatic Melanoma
Source: PLoS One. 2010 Sep 15;5(9):e12711. doi: 10.1371/journal.pone.0012711 (PMC2939876; doi:10.1371/journal.pone.0012711)
Supplement: Table S3 — Time course for activation TCR signaling in fold change from baseline. (0.04 MB DOC) [file pone.0012711.s003.doc]

**Supplemental Table 3:** Time course for activation TCR signaling in fold change from baseline.

| Time | pLck | pZAP70 | pLAT |
| --- | --- | --- | --- |
| 0 min | 1 | 1 | 1 |
| 5 min | 1.08 | 1.29 | 0.96 |
| 10 min | 1.67 | 1.28 | 0.98 |
| 15 min | 1.81 | 1.58 | 1.12 |
| 30 min | 2.56 | 1.56 | 1.38 |
| 60 min | 3.46 | 1.41 | 1.17 |
| 120 min | 3.39 | 0.87 | 0.83 |
| 24 h | 3.02 | 0.72 | 0.64 |
| 48 h | 1.75 | 0.31 | 0.33 |
