# Supplementary figures and images for: Modulation of Cell Signaling Networks after CTLA4 Blockade in Patients with Metastatic Melanoma
Source: PLoS One. 2010 Sep 15;5(9):e12711. doi: 10.1371/journal.pone.0012711 (PMC2939876; doi:10.1371/journal.pone.0012711)

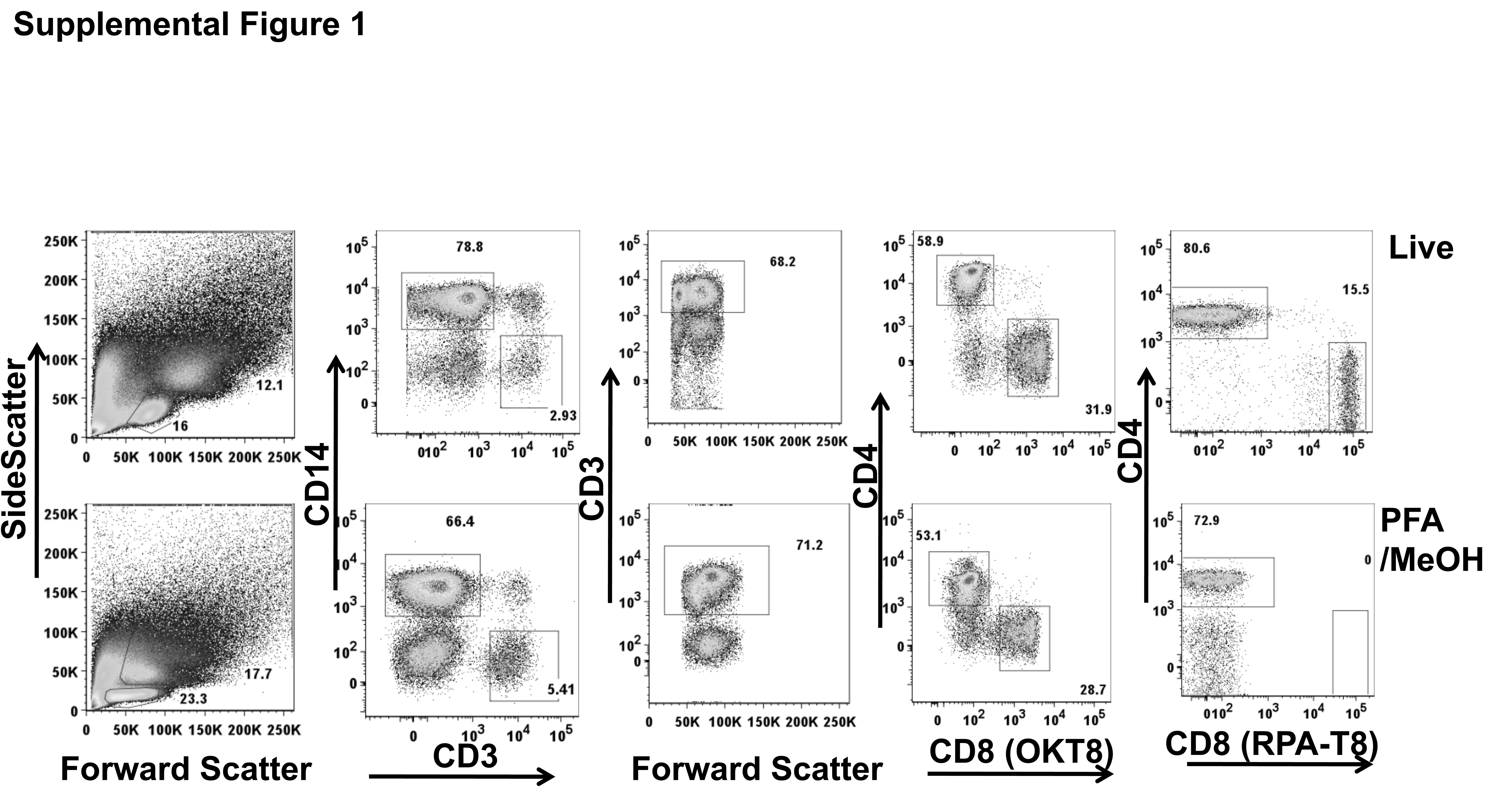

Supplement: Figure S1 — Effects of cell fixation and permeabilization on the detection of surface staining for immune cell markers. Flow cytometric identification of CD3 (UCHT1), CD4 (RPA-T4), CD14 (TüK4), and CD8 (OKT8) populations by antibody staining in fixed and permeabilized human PBMC. PBMC from patients with advanced melanoma were kept alive or fixed with 2% paraformaldehyde (PFA) and permeabilized with 90% methanol (MeOH) before staining with the respective antibodies. CD8 (RPA-T8) staining could not be identified by flow cytometry after 2% paraformaldehyde/90% methanol treatment. (3.82 MB TIF) [file pone.0012711.s004.tif]

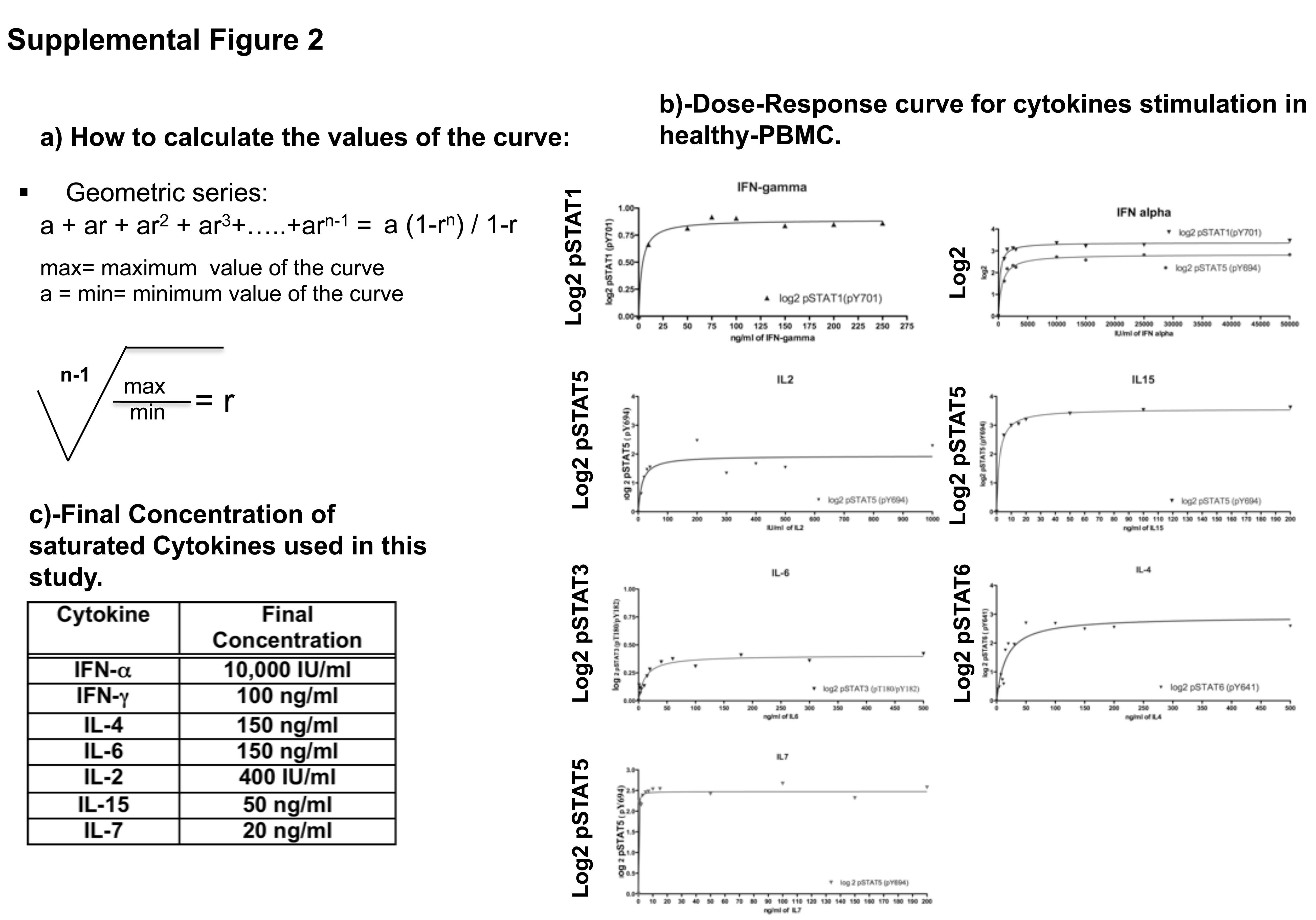

Supplement: Figure S2 — Titration of cytokines in healthy donor PBMC. a) Formula for the calculation of the geometric series. Max: maximum value of the curve, a = min: minimum value of the curve, n: numbers of points in the curve. b) Dose-response curve for IFN- alpha [readout was pSTAT1 (pY701) and pSTAT5 (pY694)]; IFN-gamma [readout pSTAT1(pY701)]; IL-2, IL-7 and IL-15 [pSTAT5 (pY694)]; IL-6 [pSTAT3 (pY705)]; IL-4 [pSTAT6(pY641)]. c) Final concentration of saturating levels of cytokines used in this study were selected from the plateau of the curve. (1.81 MB TIF) [file pone.0012711.s005.tif]

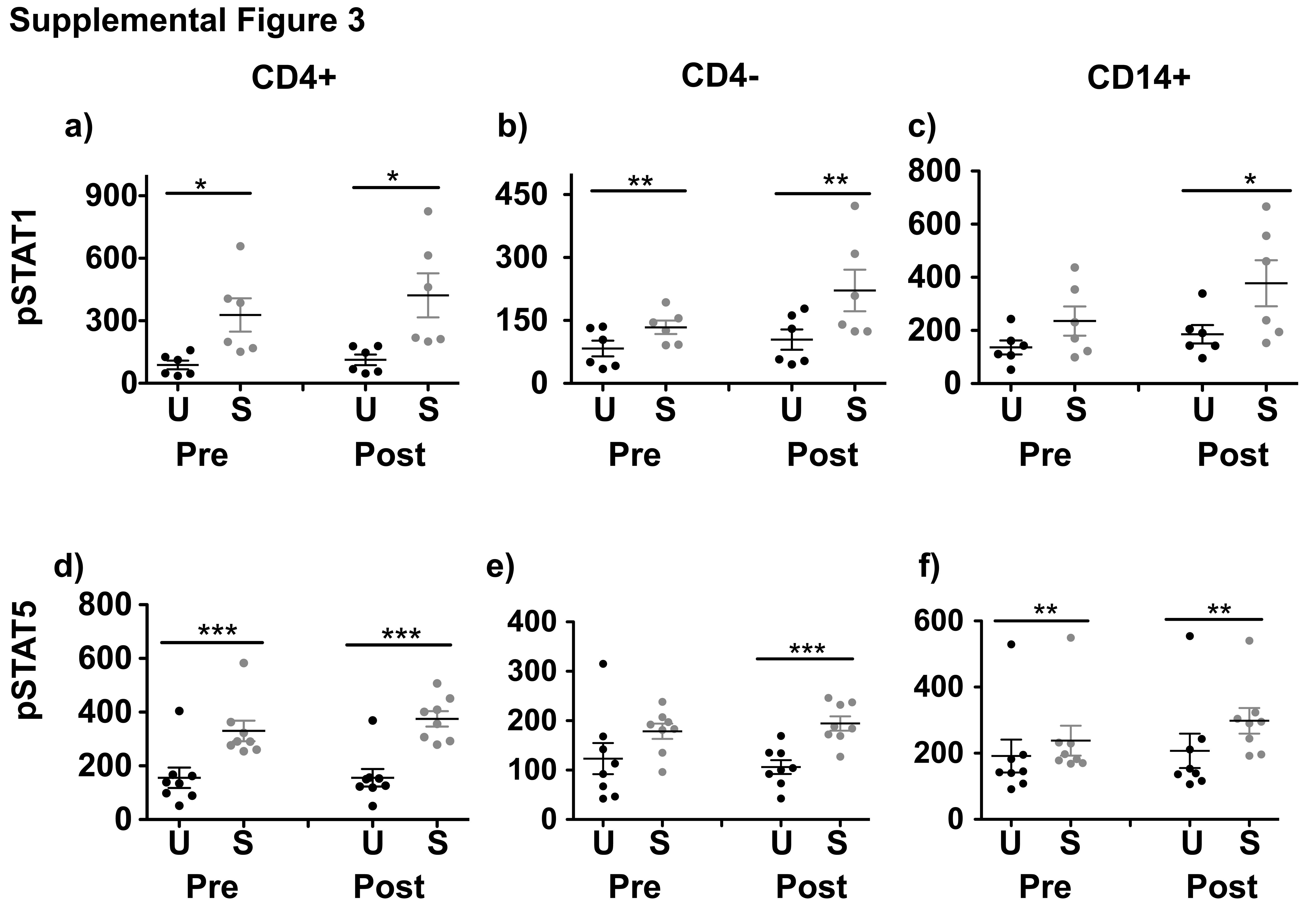

Supplement: Figure S3 — Effects of IFN-alpha cytokine stimulation on phosphorylated STAT1 and STAT5 in samples taken from patients in the combined therapy NRA study. PBMC obtained from patients before (Pre) and after (Post) receiving tremelimumab together with MART-126-35 peptide pulsed dendritic cells (DC) were stimulated with 10,000 IU/ml of IFN-alpha for 15 minutes (S) and compared to unstimulated samples (U). Labeled cells were analyzed by phosphoflow for pSTAT1 (pY701, top row) or pSTAT5 (Y694, bottom row) as described in Figure 2. a and d) CD4+ CD3+ T helper cells. b and e) CD4-CD3+ cytotoxic T lymphocytes (CTLs). c and f) CD14+ monocytes. Statistically significant results are denoted with asterisks as described in Figure 2. (0.74 MB TIF) [file pone.0012711.s006.tif]

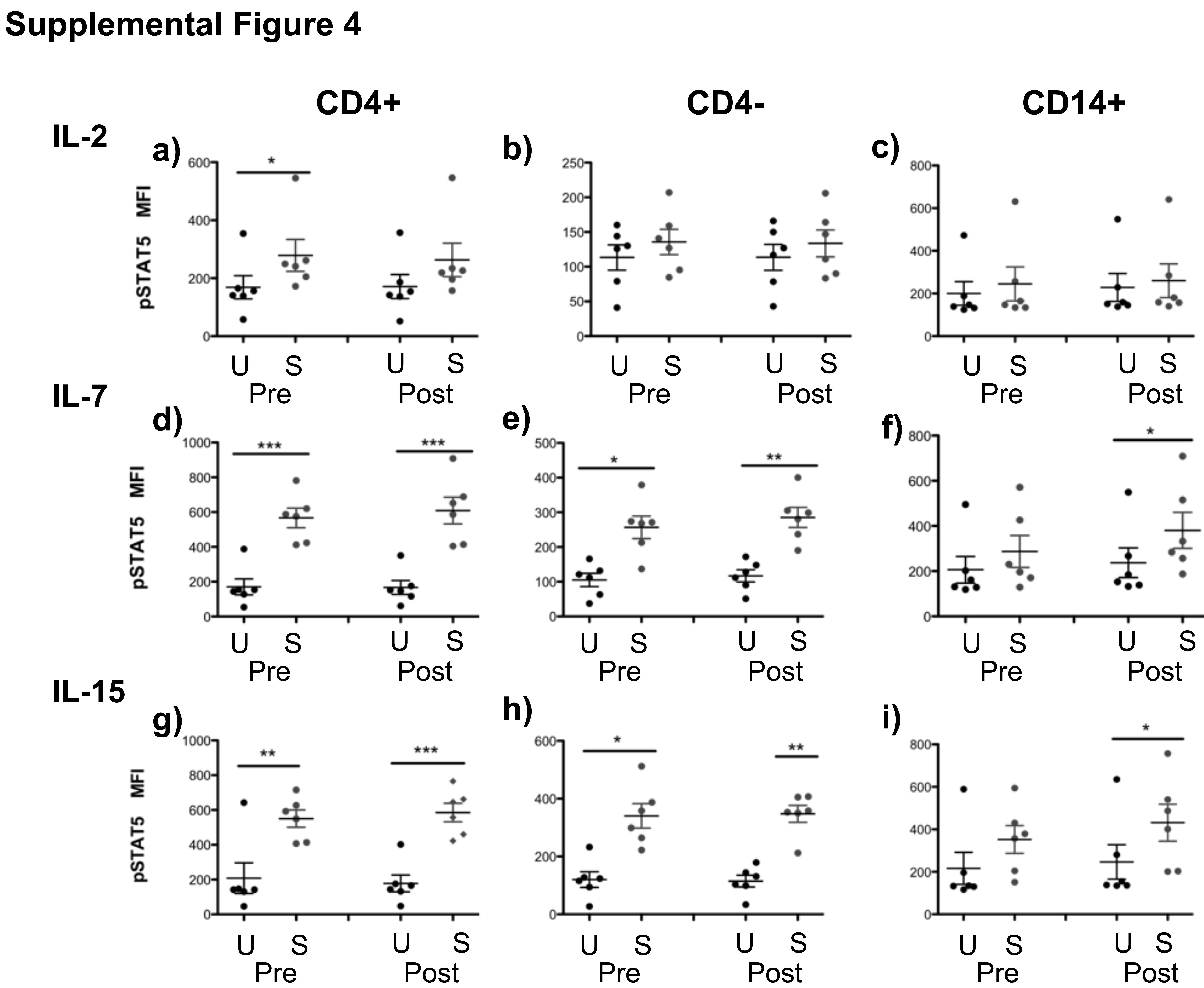

Supplement: Figure S4 — Effects of gamma chain receptor-mediated cytokine stimulation with IL-2, IL-7 and IL-15 on phosphorylated STAT5 in samples taken from patients in the combined therapy NRA study. PBMC obtained from patients before (Pre) and after (Post) tremelimumab together with MART-126-35 peptide pulsed dendritic cells (DC) were stimulated with 400 IU/ml of IL-2 (top row), 20 ng/ml of IL-7 (middle row) or 50 ng/ml of IL-15 (bottom row) for 15 minutes (S), and compared to unstimulated samples (U). Labeled cells were analyzed by phosphoflow for pSTAT5 (Y694) as described in Figure 2. a, d and g) CD4+CD3+ T helper cells. b, e and h) CD4-CD3+ cytotoxic T lymphocytes (CTLs). c, f and i) CD14+ monocytes. Statistically significant results are denoted with asterisks as described in Figure 2. (2.22 MB TIF) [file pone.0012711.s007.tif]

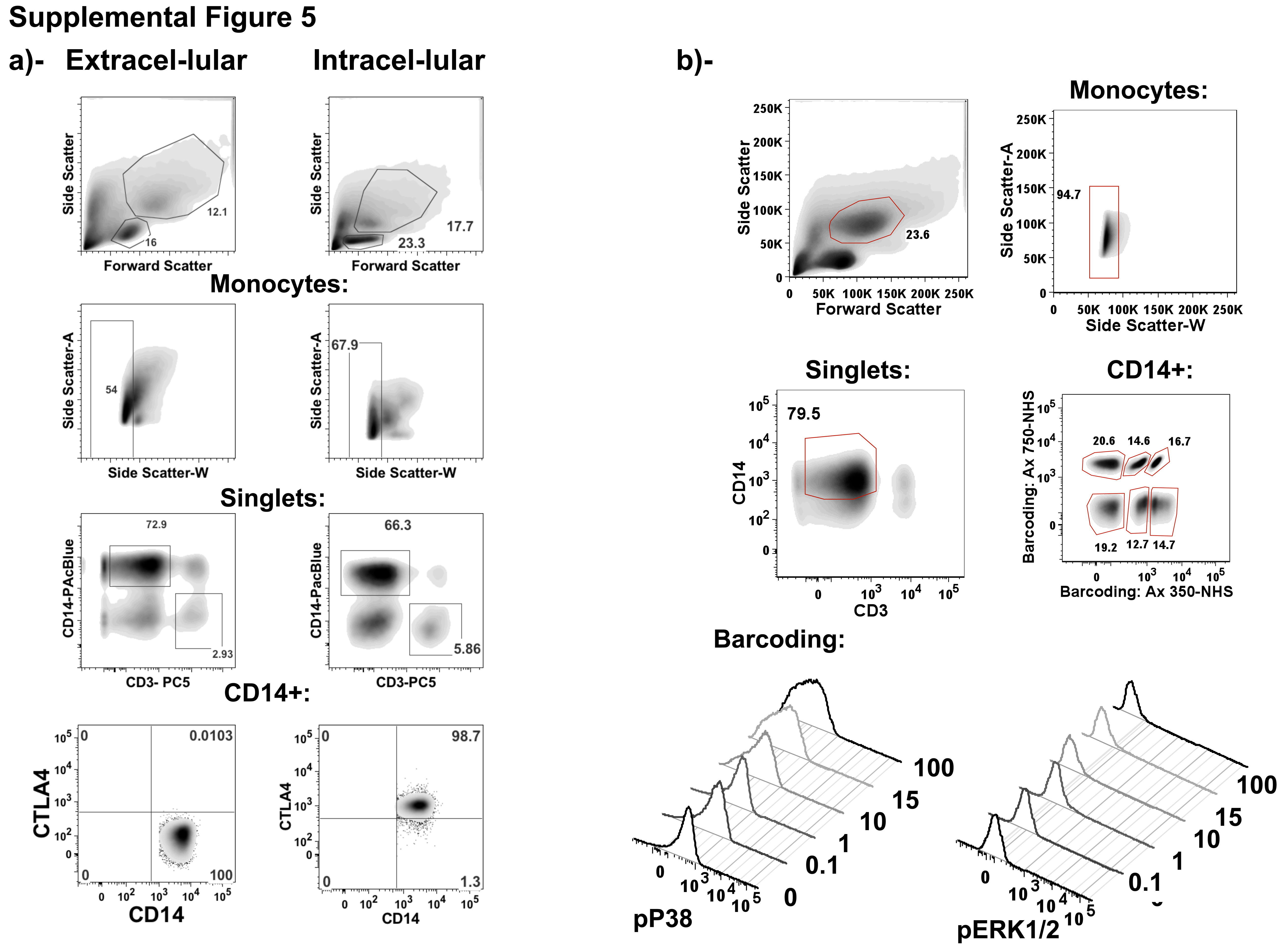

Supplement: Figure S5 — In vitro monocyte experiments. a) Representative results of extracellular and intracellular CTLA4 expression in monocytes. After gating monocytes by morphology, single cells were gated as CD14+CD3-. b) Gating strategy for phosphorylation experiments. After gating on monocytes by morphology, single cells were gated as CD14+CD3-. Six populations of cells, each one exposed to a different concentration of tremelimumab for 48 hours, were analyzed simultaneously based on fluorescent dye barcoding with Ax350-NHS and Ax-750-NHS. Each one of the differently labeled groups represents cells treated with increasing concentrations of tremelimumab in which intracellular phosphoproteins were analyzed by mean fluorescence intensity (MFI). (2.32 MB TIF) [file pone.0012711.s008.tif]
